# Effects of Polysaccharide Elicitors on Secondary Metabolite Production and Antioxidant Response in *Hypericum perforatum* L. Shoot Cultures

**DOI:** 10.1155/2014/609649

**Published:** 2014-12-11

**Authors:** Sonja Gadzovska Simic, Oliver Tusevski, Stéphane Maury, Alain Delaunay, Claude Joseph, Daniel Hagège

**Affiliations:** ^1^Laboratoire de Biologie des Ligneux et des Grandes Cultures, UPRES EA 1207, UFR-Faculté des Sciences, Université d'Orléans, rue de Chartres, BP 6759, 45067 Orléans Cedex 2, France; ^2^Institute of Biology, Faculty of Natural Sciences and Mathematics, University of Ss. Cyril and Methodius, P.O. Box 162, 1000 Skopje, Macedonia

## Abstract

The effects of polysaccharide elicitors such as chitin, pectin, and dextran on the production of phenylpropanoids (phenolics and flavonoids) and naphtodianthrones (hypericin and pseudohypericin) in *Hypericum perforatum* shoot cultures were studied. Nonenzymatic antioxidant properties (NEAOP) and peroxidase (POD) activity were also observed in shoot extracts. The activities of phenylalanine ammonia lyase (PAL) and chalcone-flavanone isomerase (CHFI) were monitored to estimate channeling in phenylpropanoid/flavonoid pathways of elicited shoot cultures. A significant suppression of the production of total phenolics and flavonoids was observed in elicited shoots from day 14 to day 21 of postelicitation. This inhibition of phenylpropanoid production was probably due to the decrease in CHFI activity in elicited shoots. Pectin and dextran promoted accumulation of naphtodianthrones, particularly pseudohypericin, within 21 days of postelicitation. The enhanced accumulation of naphtodianthrones was positively correlated with an increase of PAL activity in elicited shoots. All tested elicitors induced NEAOP at day 7, while chitin and pectin showed increase in POD activity within the entire period of postelicitation. The POD activity was in significantly positive correlation with flavonoid and hypericin contents, suggesting a strong perturbation of the cell redox system and activation of defense responses in polysaccharide-elicited *H. perforatum* shoot cultures.

## 1. Introduction

*Hypericum perforatum *L. (St. John's wort) is a herbaceous perennial plant that has received considerable interest in North America and Europe due to its medicinal properties. This plant produces several types of biologically active compounds, including naphtodianthrones (hypericin and pseudohypericin), prenylated acylphloroglucinols (hyperforin and adhyperforin), flavonoids (quercetin, hyperoside, rutin, and quercitrin), xanthones (1,3,6,7-tetrahydroxyxanthone), and essential oil rich in sesquiterpenes [1]. In phytomedicine,* Hypericum* extracts have a wide range of pharmacological properties, including wound healing [2], anti-inflammatory [3], antitumoral [4], antiviral [5], antimicrobial [6], antioxidant [7], and apoptosis-inducing activities [8]. The most important use of* H. perforatum* comprises symptomatic treatment of mild-to-moderate depression [9] and recently good perspectives emerged in the field of major depressive disorder [10]. Taking into account these pharmacological activities,* H. perforatum* preparations represent one of the leading herbal dietary supplements worldwide [11].

As a consequence of great commercial potential of* H. perforatum* and necessity to maintain the massive market demand, a great effort has been directed toward management of field cultivation of this plant in order to maximize yield and quantity of metabolites with therapeutic properties [12]. Unfortunately, the content of secondary metabolites in field-cultivated* H. perforatum* is notably affected by genetic, physiological, ecological, and environmental factors [13, 14]. All these factors are responsible for qualitative and quantitative variations of bioactive metabolites in commercially produced* H. perforatum* pharmaceutical preparations [15]. Therefore, the establishment of* in vitro* protocols for cultivation of* H. perforatum* with standardized concentrations of bioactive compounds is required for commercial and research applications [16].

Plant cell culture technology has recently shown great priority as an alternative to the whole plant system for producing commercially important bioactive products [17]. In addition, many studies have been focused on methods that increase the productivity of plant* in vitro* cultures, such as medium optimization, cell line selection, cell immobilization, precursor feeding, metabolic engineering, and elicitation [18]. Among these manipulation techniques, elicitation represents a very attractive strategy for enhancement of secondary metabolite production in plant culture systems. Recent studies have shown that plant bioactive compounds can be transiently produced in high quantities in response to external stimuli or elicitors [17, 19]. The use of biotic or abiotic elicitors to stimulate product formation has become an important strategy and has been very useful in reducing the process time required to attain high product concentrations and increased volumetric productivity [20]. Abiotic elicitors predominantly consist of physical and chemical stresses such as metal ions, other inorganic compounds, or even UV radiation and electric current [21]. On the other hand, biotic elicitors are substances with biological origin such as polysaccharides derived from plant cell walls, for example, pectin, pectic acid, and cellulose, while those of microorganism origin are cell wall components, like chitin, chitosan, or glucans [21, 22].

Poly- or oligosaccharides are signaling molecules within elicitation pathways that have been intensively studied because these compounds can induce similar plant defense response to pathogen invasion [19]. The mechanisms by which plant cells perceive such microbial glucans and oligosaccharides are not fully understood, although a growing body of evidence has verified the positive effects of these elicitors on the production of secondary metabolites in various plant species [23–26].* H. perforatum in vitro* cultures have been the subject of many research studies focused on enhancement of naphtodianthrone and phenylpropanoid production upon treatment with various elicitors [27–34]. In this context, only a few studies have been carried out to investigate the effects of polysaccharide elicitors on naphtodianthrone production in* H. perforatum in vitro* cultures [27, 31]. Namely, the effect of mannan, *β*-1,3-glucan, and pectin on the production of naphtodianthrones in* H. perforatum* shoot cultures has been studied [27]. Similarly, Vardapetyan et al. [31] evaluated the effects of mannan and *β*-1,3-glucan on hypericin and pseudohypericin production in* H. perforatum* callus cultures. Nevertheless, the coordination of the production of naphtodianthrones (hypericin and pseudohypericin) with other secondary metabolites (phenolics and flavonoids) in* H. perforatum* shoot cultures upon treatment with polysaccharide elicitors is rather limited.

In this study, the effects of different polysaccharide elicitors, for example, chitin (CHI), pectin (PEC), and dextran (DEX), on phenylpropanoid (phenolics and flavonoids) and naphtodianthrone (hypericin and pseudohypericin) production in* H. perforatum* shoot culture were investigated. The activities of two key enzymes of the phenylpropanoid/flavonoid pathways, phenylalanine ammonia lyase (PAL) and chalcone-flavanone isomerase (CHFI), were also evaluated to examine the potential relationship between different metabolic pathways. Nonenzymatic antioxidant properties (NEAOP) and peroxidase (POD) activity were also monitored to evaluate the possible correlation between secondary metabolite production and antioxidant defence mechanisms of elicited cells.

## 2. Material and Methods

### 2.1. Plant Material

Seeds from* H. perforatum *were collected from plants growing in a natural population in the National Park Pelister at about 1394 m. Voucher specimen number (060231) of* H. perforatum* is deposited in the Herbarium at the Faculty of Natural Sciences and Mathematics, University of Ss. Cyril and Methodius, Skopje, Macedonia (MKNH). Seeds were washed with 70% ethanol for 30 sec, surface sterilized with 1% NaOCl for 15 min, rinsed 3 times in sterile deionized water [30], and cultured on MS macro- and oligoelements [35], B_5_ vitamin solution [36], supplemented with 3% sucrose and solidified with 0.7% agar. No growth regulator was added. The medium was adjusted to pH 5.8 before autoclaving (20 min at 120°C).* In vitro* cultures were maintained in a growth chamber at 25 ± 1°C under a photoperiod of 16 h light, irradiance at 50 *μ*mol*·*m^2^ *·*s^−1^, and 50 to 60% relative humidity.

Apical segments containing 2 to 4 leaves were excised from 2-3-week-old* in vitro* grown plant and used as explants to establish shoots. The explants were cultured in 100 mL flasks containing 40 mL of MS/B_5_ medium with 3% sucrose, 200 mg*·*L^−1^ casein enzymatic hydrolysate and supplemented with 0.5 mg*·*L^−1^ benzyladenine. Multiplication of isolated apical segments from* in vitro* germinated seedlings was obtained after 2-3 weeks of culture. After 3 weeks, shoot apices (20–25 mm long) were isolated from multiplied shoots and subcultured every month.

### 2.2. Elicitor Preparation and Treatments

Pectin (PEC) and dextran (DEX) solutions (Sigma-Aldrich Division, France) were prepared in sterile distilled water and sterilized on a 0.22 *μ*m Millipore filter. Stock solution of crab shell chitin (CHI) was prepared in glacial acetic acid (1 g in 2 mL) by adding dropwise at 60°C for a period of 15 min and the final volume was made up to 100 mL. The pH of the elicitor solutions was adjusted to 5.8 with 1 M NaOH before autoclaving at 120°C for 20 min [37]. Filter-sterilized elicitor solutions were added to the autoclaved culture medium individually. Treatments of* H. perforatum* shoot cultures with CHI, PEC, and DEX (100 mg*·*L^−1^) were performed after the 3rd subculture. For the control set of cultures, distilled water substituted the elicitors. Treated and control shoots were then harvested on days 7, 14, and 21 of postelicitation, frozen in liquid nitrogen or lyophilized, and stored at −80°C, until analysis.

### 2.3. Extraction and Quantification of Secondary Metabolites

Phenolic compounds extraction and quantification were performed as previously reported [32, 34]. Briefly, phenolic compounds were extracted from freeze-dried lyophilized and powdered plant material (0.2 g) with 80% (v/v) methanol in ultrasonic bath for 30 min at 4°C.

Total phenolic (TP) contents were determined when methanolic shoot extracts were mixed with Folin-Ciocaltea reagent (Carlo Erba Reagenti, Rodano, Italy) and 0.7 M Na_2_CO_3_ [38]. Samples were incubated for 5 min at 50°C and then cooled for 5 min at room temperature. Absorbance was measured spectrophotometrically at 765 nm. The concentration of TP was calculated using gallic acid as a standard. The results were expressed as mg gallic acid equivalents (GAE) per g dry mass (mg GAE*·*g^−1^ DM).

Total flavonoid (TF) contents were determined by using a method described by Makris et al. [39]. An aliquot of appropriately diluted (1 : 10–1 : 100, v/v) extract was mixed with 5% NaNO_2_ and allowed to react for 5 min. Following this, 10% AlCl_3_ was added and the mixture stood for further 5 min. Finally, to the reaction mixture, 1 M NaOH and distilled water were added. Absorbance was measured spectrophotometrically at 510 nm. TF content was calculated from a calibration curve using catechin as a standard. The results were expressed as mg catechin equivalents (CE) per gram dry mass (mg CE*·*g^−1^ DM).

### 2.4. Nonenzymatic Antioxidant Properties (NEAOP) Assay by *β*-Carotene Bleaching Method

Nonenzymatic antioxidant properties (NEAOP) of methanolic extracts were estimated by using linoleic acid-*β*-carotene oxidation method adapted from Marron et al. [40]. A linoleic acid-*β*-carotene emulsion was prepared by mixing 10 mg of linoleic acid with 750 *μ*L of 0.2 mg*·*mL^−1^ chloroformic *β*-carotene solution and 100 mg of Tween 40 (polyoxyethylene sorbitan monopalmitate). Chloroform was evaporated under nitrogen flow for 10 min. The resulting mixture was adjusted to 25 mL with distilled water and shaken for 10 seconds. The reaction mixture was prepared as follows: 10 *μ*L of extract was adjusted with 15 *μ*L 80% (v/v) methanol and 225 *μ*L of linoleic acid-*β*-carotene emulsion was added. The mixture was heated to 50°C. The control consisted of 25 *μ*L of 80% (v/v) methanol and 225 *μ*L of linoleic acid-*β*-carotene emulsion. Absorbance was measured at 470 nm every 15 minutes for 45 minutes. Results were computed as the ratio of *β*-carotene protection of the extract to the control (80% methanol). NEAOP was calculated using the following formula: NEAOP = ((*B* − *A*)/*B*) × 100, where *A* is variation of absorbance of samples between 0 and 45 min; *B* is variation of absorbance of control between 0 and 45 min.

### 2.5. Enzyme Extraction and Assays

The extraction procedure for determination of antioxidant enzyme assays was based on the method as previously described by Gadzovska et al. [32]. The enzyme extract was prepared by homogenizing 1 g of frozen sample in 2 mL 0.1 M KH_2_PO_4_/K_2_HPO_4_ buffer at pH 8.0, containing 2 mM ethylenediamine tetra-acetic acid (EDTA), 1.4 mM *β*-mercaptoethanol, and 1% (w/v) polyvinylpyrrolidone (PVP). The homogenate was centrifuged at 13000 rpm for 20 min at 4°C. The supernatant was collected for determination of protein content and enzyme assays. Protein contents in enzyme extracts were performed with a Bio-Rad Protein Assay Reagent. Bovine serum albumin (BSA) was used as a standard.

Peroxidase (POD) assay was based on a method described by González et al. [41]. The reaction mixture contained 0.1 M NaH_2_PO_4_/Na_2_HPO_4_ buffer (pH 6.0), 20 mM guaiacol solution, 0.1% (v/v) H_2_O_2_, and diluted enzyme extract (1 : 10, v/v). The absorbance was monitored in 40 s for a period of 1 min and 20 s at 420 nm. The rate of change in absorbance per minute was used to quantify POD activity using the molar extinction coefficient of the oxidized product tetraguaiacol *ε*_420_ = 6400 M^−1^ *·*cm^−1^. POD specific activity was determined as the increase in absorbance and expressed in nkat*·*mg^−1^ proteins.

Phenylalanine ammonia lyase (PAL) assay was determined according to Gadzovska et al. [32, 34]. The reaction mixture contained 2% (w/v) solution of L-phenylalanine in 50 mM Tris-HCl at pH 8.8 and enzyme extract. Enzyme assay mixtures were incubated at 40°C for 60 min. PAL activity was determined by measuring the rate of formation of* trans*-cinnamic acid as increase in absorbance at 290 nm. Molar extinction coefficient of cinnamate was *ε*_290_ = 19600 M^−1^ *·*cm^−1^. The PAL activity was expressed in pkat*·*mg^−1^ proteins.

Chalcone-flavanone isomerase (CHFI) enzyme assay was based on the method described by Gadzovska et al. [32, 34]. CHFI was assayed in 60 mM KH_2_PO_4_/K_2_HPO_4_ buffer at pH 8.0, containing 50 mM KCN to inhibit peroxidase activity. Reaction was initiated by mixing enzyme extract and 2′,4,4′,6-tetrahydroxychalcone. Enzyme assay mixture was incubated at 30°C for 45 min. The kinetics of the reaction was monitored by measuring the decrease in absorbance at 400 nm. Molar extinction coefficient of 2′,4,4′,6-tetrahydroxychalcone was *ε*_400_ = 33113 M^−1^ *·*cm^−1^. The CHFI activity was expressed in pkat*·*mg^−1^ proteins.

### 2.6. High Performance Liquid Chromatography and Electrospray Ionization Mass Spectrometry (HPLC/ESI-MS) Analysis of Naphtodianthrones

Hypericin and pseudohypericin extractions were performed as described by Gadzovska et al. [30]. A Shimadzu LC-6A liquid chromatograph equipped with a fluorescence detector Shimadzu RF-535 (*λ*_exc_ = 236 nm and *λ*_em_ = 592 nm) was used for end-point detection. HPLC analyses were carried out at 25°C on a Hypersil reversed-phase C_18_ column (150 × 4.6 mm, 5 *μ*m, Interchim, France). Mobile phase A was triethylammonium acetate buffer (0.01 M) at pH 7.0, and phase B was mixture of methanol and acetonitrile (5 : 4, v/v). The analyses followed linear gradient program with a flow rate of 1.5 mL*·*min^−1^ with 20 *μ*L injected volume. Linear gradient combinations were started with 40% *A* and 60% *B* (0–3 min), 8% *A* and 92% *B* (4–9 min), and 0% *A* and 100% *B* (10 min). Total run time was 10 min. Standard solutions of hypericin (0–100 *μ*g*·*mL^−1^) were prepared from pure commercially available standard of hypericin (Sigma, France). Pseudohypericin was isolated from plant extracts and purified onto semipreparative Nucleosil C_18_ column (250 × 10 mm, 5 *μ*m, Interchim, France). Standard solutions of pseudohypericin were prepared in concentration range of 0 to 100 *μ*g*·*mL^−1^. Chromatograms were performed at 590 nm. All reagents were HPLC grade (Merck, Germany).

As previously described by Gadzovska et al. [30], mass spectra of naphtodianthrones were acquired using a LCQ Deca mass spectrometer, equipped with an atmospheric pressure chemical ionization source (Thermo-Finnigan). The instrument was operated in the negative ion mode, scanning from* m/z* 150 to 600. Operating conditions were sheath gas: 65 psi; auxiliary gas (nitrogen): 10 psi; ESI needle voltage: 4.5 kV; capillary temperature: 250°C; and capillary voltage: −12 V. For multiple MS (MS^2^) spectra of selected precursor ions, the activation energy was 53% for hypericin and 50% for pseudohypericin. Compounds were introduced to the fused silica-lined ESI needle by syringe pump at 5 *μ*L*·*min^−1^ flow rates. Data acquisition and processing were performed with Xcalibur software (version 1.2).

### 2.7. Statistical Analyses

The experiments were independently repeated twice under the same conditions and all analyses were performed in triplicate. Error bars of graphs show the standard deviation of mean value (± SD). The statistical analyses were performed with the SPSS statistical software program (SPSS version 11.0.1 PC, IL, USA). All statistical tests were considered significant at *P* < 0.05.

## 3. Results and Discussion

### 3.1. Phenylpropanoid and Naphtodianthrone Production

**Figure 1:**
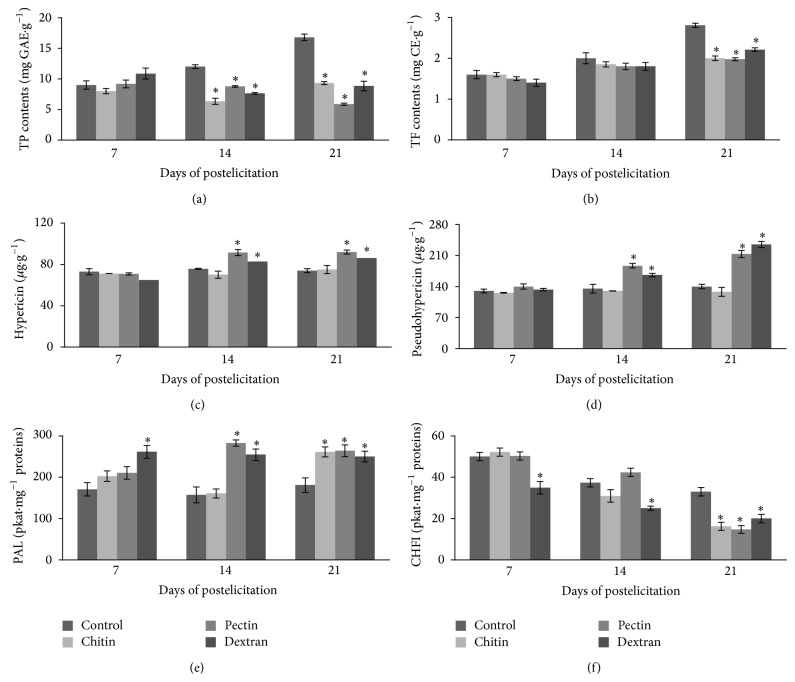
Effects of polysaccharide elicitors on the production of (a) phenolics, (b) flavonoids, (c) hypericin, (d) pseudohypericin, and enzymatic activities of (e) phenylalanine ammonia lyase and (f) chalcone-flavanone isomerase in* Hypericum perforatum* shoot cultures. TP: total phenolics; TF: total flavonoids; PAL: phenylalanine ammonia lyase; CHFI: chalcone-flavanone isomerase. Level of significance is ^*^ *P* < 0.05.

The effects of polysaccharide elicitors on TP and TF production in shoot cultures of* H. perforatum* are shown in Figure 1. With regard to control shoot cultures, the levels of TP were linearly increased during the postelicitation: from 9 mg GAE*·*g^−1^ at the beginning to 17 mg GAE*·*g^−1^ DM at day 21 of culture. The same linear trend in increase of TF accumulation was observed in control shoots: the amounts of TF were 1.6 mg CE*·*g^−1^ at day 7 and reached about 2.8 mg CE*·*g^−1^ DM after day 21 of postelicitation. After treatment with tested polysaccharides, no elicitation was ascertained at day 7, but, on the contrary, significant inhibition in TP production was observed at days 14 and 21 (Figure 1(a)). During this period of postelicitation, treatment with CHI and DEX resulted in heterogeneous pattern of TP production in shoot cultures, with levels of about 1.6- to 1.9-fold less than respective controls. It is noteworthy that PEC showed the strongest inhibitory effect on TP content in elicited shoots (about 3-fold) compared to control ones at day 21. Similar results of TF contents upon polysaccharide elicitation in shoot cultures were observed (Figure 1(b)). In this view, TF production in shoots treated with CHI, PEC, and DEX was significantly suppressed during the late phase of postelicitation (day 21). These elicitors showed similar rate of TF production in treated shoot cultures, with significantly lower amounts (from 1.3- to 1.4-fold) than corresponding controls. A significant correlation between TP and TF contents (*r* = 0.571; ^*^ *P* < 0.05) was found demonstrating that flavonoids are dominant group of phenolic compounds estimated in elicited shoot cultures (Table 1).

Several studies have been focused on the production of phenolics and flavonoids in* H. perforatum in vitro* cultures as a defense response to various stress factors [32, 34, 42–44]. With respect to phenylpropanoid production in* H. perforatum* shoot cultures, we have already reported that the levels of TP and TF were unchanged or decreased upon treatment with salicylic acid (50–250 *μ*M) as an elicitor [34]. Additionally,* Agrobacterium rhizogenes* as a bacterial elicitor stimulated the production of TP, while TF were simultaneously decreased in* H. perforatum* adventitious shoot cultures [42]. On the other hand, our previous results for phenylpropanoid production in* H. perforatum* cell cultures showed that chemical elicitors (jasmonic acid and salicylic acid) and bacterial elicitors (*A. tumefaciens* and* A. rhizogenes*) stimulated the production of TP and TF [32, 34, 45]. Additionally, Cui et al. [43] reported an increase in TP content in* H. perforatum* root cell cultures upon sucrose-induced osmotic stress. These results indicated that* H. perforatum* shoots did not give clear-cut answer to exogenously applied elicitors, but cells showed remarkably fast and strong response in the production of secondary metabolites. In this context,* H. perforatum* cell suspensions have a higher rate of metabolism than shoots because the initiation of cell growth in culture leads to fast proliferation of cell biomass and stimulated phenylpropanoid biosynthetic pathway [32, 34, 45]. Shoot cultures provide little possibility for alteration of phenolic profile (e.g., by treatments with elicitors), which is in contrast with cell suspensions. The differences may be associated with the fact that shoot cultures consist of various differentiated cell types, whereas cell cultures provide more homogeneous systems.

In this study, hypericin and pseudohypericin as naphtodianthrone derivatives were determined by HPLC analysis in shoot extracts. In control shoot cultures, the levels of hypericin (Figure 1(c)) and pseudohypericin (Figure 1(d)) were similar and did not show variation along the culture period (about 74 *μ*g g^−1^ DW and 135 *μ*g g^−1^ DW, resp.). Accordingly, the content of pseudohypericin was almost doubled compared to hypericin level, as reported in shoot cultures of* H. perforatum* [27]. The treatments of shoots with polysaccharide elicitors did not show significant influence on hypericin and pseudohypericin production during the first 7 days of postelicitation. Among the tested elicitors, only CHI had no stimulatory effect on the naphtodianthrone production during the entire period of postelicitation. Several reports showed that CHI was less effective elicitor of secondary metabolite production than other biotic elicitors [46, 47]. Outgoing results showed that production of both naphtodianthrones was stimulated by PEC and DEX treatments during the late phase of postelicitation (days 14 and 21). A maximum of hypericin contents in treated shoots was found after 14 days of postelicitation and thereafter slightly decreased. At days 14 and 21 of treatment with PEC, hypericin contents were about 1.2-fold higher compared to control, while DEX stimulated hypericin production to a lesser degree (Figure 1(c)). The production of pseudohypericin was significantly stimulated by treatments with PEC and DEX at day 14 (about 1.4- and 1.2-fold, resp.) compared to control level. At day 21 (Figure 1(d)), treatments with DEX and PEC enhanced production of pseudohypericin (about 1.7- and 1.5-fold, resp.). Linear and positive correlation was found between the contents of hypericin and pseudohypericin (Table 1) in shoot cultures (*r* = 0.854; ^***^ *P* < 0.001). Results from this study indicate that production of hypericin and pseudohypericin in* H. perforatum* shoot cultures could be modified with application of polysaccharide elicitors such as PEC and DEX.

As far as the authors are aware, naphtodianthrone production has been intensively studied in cell, tissue, and organ cultures of* H. perforatum* [27, 29, 48, 49]. A detailed study of Pasqua et al. [50] on the accumulation of bioactive metabolites in* H. perforatum* undifferentiated cell cultures, compared with shoot cultures, clearly demonstrated that organ differentiation is necessary to obtain hypericins. In addition, we have already reported a relationship between the biosynthesis of naphtodianthrones and the number of dark glands on the leaves in* H. perforatum* shoot cultures [51]. A number of studies have been adopted to improve the production of hypericins in various* Hypericum in vitro* cultures by using different biotic or abiotic elicitors [27–29, 32–34, 45, 48, 52]. Although* H. perforatum in vitro* cultures are known to produce naphtodianthrones, there has been little work to investigate whether these compounds are inducible by elicitation with polysaccharides. In this view, naphtodianthrone accumulation by polysaccharide elicitors has been observed in* H. perforatum* shoot cultures [27] and calli [31]. Kirakosyan et al. [27] demonstrated that mannan stimulated pseudohypericin and hypericin production in shoot cultures, while *β*-1,3-glucan and PEC slightly enhanced pseudohypericin production but had no effect on hypericin production. On the other hand, yeast extract showed inhibitory effect on either hypericin or pseudohypericin production. Similarly, Vardapetyan et al. [31] showed that mannan stimulated biosynthesis of both hypericin and pseudohypericin, while *β*-1,3-glucan had positive effect only on pseudohypericin contents in callus cultures. These authors have proposed mannan as a strong elicitor of naphtodianthrone production in* H. perforatum in vitro* cultures. Present results showed that PEC and DEX possess greater stimulating action on naphtodianthrone production, while CHI did not show elicitor activity. In addition, PEC and DEX showed more universal effects on enhanced production of hypericin and pseudohypericin. Therefore, we suggest that PEC and DEX could mimic stress conditions and the enhancement in levels of hypericin and pseudohypericin appears to have a potential role in defense strategy of* H. perforatum *shoots.

The activities of two key enzymes, PAL and CHFI, in elicited cells were also monitored to estimate general channeling in the phenylpropanoid/flavonoid pathways. In control shoots, PAL activity remained relatively stable (about 170 pkat mg^−1^ proteins) during the cultivation period of day 21. It is worth noting that PAL activity was significantly increased (about 1.5-fold) in the presence of DEX during the entire period of postelicitation compared to control shoots (Figure 1(e)). With respect to CHI and PEC, the increase of PAL activity was observed only at days 14 and 21. At last, the maximum of PAL activity (about 1.8 fold) was found in shoots treated with PEC at day 14 of postelicitation. A significant positive correlation between PAL activity and hypericin and pseudohypericin production (Table 1) in elicited shoots was noticed (*r* = 0.547; ^*^ *P* < 0.05; *r* = 0.562; ^*^ *P* < 0.05, resp.). In the present study, an elevation of PAL activity with simultaneous increase in hypericin and pseudohypericin production upon addition of polysaccharides indicated that these elicitors could be effective in activation of naphtodianthrone pathway. The biosynthetic pathway leading to hypericins starts with the condensation of one molecule of acetyl-CoA with seven molecules of malonyl-CoA to form an octaketide chain that subsequently undergoes cyclizations and decarboxylation to form emodin anthrone. These reactions are carried out by two polyketide synthases (HpKS1 and HpKS2) having octaketide synthase activity [53]. Emodin anthrone further oxidized to emodin, which undergoes oxidative dimerization to finally form hypericin [54]. All these reactions have been suggested to be catalyzed by a phenolic coupling protein HYP-1 [55]. Košuth et al. [56] showed that there is no difference in hyp-1 expression between leaf margins that contained hypericin accumulating dark glands and leaf interior parts free of dark glands. Continuing the research on this material, Karppinen [57] considered that HYP-1 protein is mobile but its target is not the dark glands. In agreement, we found that Hyp-1 mRNA could be detected in cells and that salicylic acid elicitation did not influence its accumulation [34]. Furthermore, Michalska et al. [58] suggested that the function of HYP-1 may also be closer to the storage or transport of hypericin than to biosynthesis. These observations suggested that different unknown genes might be responsible for the biosynthesis of hypericins in different places.

In this study, a high relationship between naphtodianthrone production and PAL activity was observed in* H. perforatum *shoots upon polysaccharide elicitation. The coordinated induction of PAL and naphtodianthrone production was already reported in our previous studies for* H. perforatum *cell cultures upon elicitation with jasmonic acid and salicylic acid [32, 34]. Similarly, Xu et al. [59] found that sodium nitroprusside stimulated the PAL activity and hypericin production in* H. perforatum* suspended cells. Recent study of Klejdus et al. [60] showed that AIP (2-aminoindane-2-phosphonic acid) as an inhibitor of PAL activity leads to decrease in phenolic accumulation, in particular naphtodianthrones (HYP and PHYP) in* H. perforatum* shoot cultures. As basic structural unit of hypericin is emodin anthrone, the search for possible biosynthetic routes for anthraquinone synthesis revealed the existence of an alternative pathway, through chorismate/*o*-succinylbenzoic acid pathway [61, 62]. Therefore, we can consider that stimulated PAL activity upon polysaccharide elicitation might trigger the defense responses of* H. perforatum* shoots through activation of synthetic pathway of naphtodianthrones.

Even though PAL is the key regulatory enzyme leading to the formation of a wide range of phenylpropanoid metabolites [63], it is well-known that CHFI activity is essential for the biosynthesis of flavonoid defense compounds such as flavones, flavonols, anthocyanins, and condensed tannins [64]. Phenylpropanoid/flavonoid biosynthetic pathways are among the most frequently observed metabolic activities that are induced upon treatment of plant tissue or cultured cells with different elicitors. The enhancement of phenylpropanoid production upon biotic stress is usually associated with a rapid, transient increase in activities of PAL and CHFI, two key enzymes of the phenylpropanoid/flavonoid pathway [44]. However, present results demonstrated inhibition in CHFI activity in polysaccharide-elicited cells (Figure 1(f)). Even though DEX showed stable inhibition of CHFI activity (about 1.5-fold) during the entire period of postelicitation, the addition of CHI and PEC resulted in much higher suppression of enzyme activity (about 2-fold) only at the end of cultivation period (day 21). As CHFI regulates the flavonoid pathway, a lower level of TF compounds in polysaccharide-treated shoots is probably due to the inhibition of this key enzyme.

This is the first study where the accumulation of naphtodianthrones and flavonoids was differently affected upon polysaccharide elicitation, although Hillwig et al. [65] proposed that these two groups of metabolites derived from similar polyketide pathways. The type III polyketides result from the condensation of 3 malonyl-CoA molecules and various precursor substrates derived from branch chain shikimate-derived phenylpropanoids (flavonoids) and acetyl-CoA (naphtodianthrones). Divergent type III polyketide synthases have evolved to create this wide variety of polyketides. Therefore, with regard to the effects of polysaccharide elicitors on flavonoid and naphtodianthrone production, two potential explanations are possible: (1) induction of PAL activity triggers an alternative synthetic naphtodianthrone pathway and (2) inhibition of flavonoid production due to suppressed CHFI activity indicated that hypericin biosynthesis is prioritized over flavonoids. However, there are still many open questions relating to the specificity of certain enzymes or genes for the conversation of specific compounds versus more general roles in catalyzing certain kinds of chemical reactions [66].

### 3.2. Antioxidant Response of Elicited Shoot Cultures

**Figure 2:**
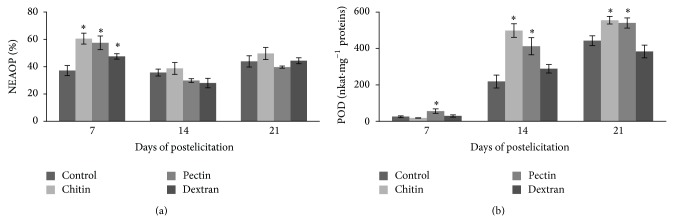
Effects of polysaccharide elicitors on (a) nonenzymatic antioxidant properties and (b) peroxidase activity in* Hypericum perforatum* shoot cultures. NEAOP: nonenzymatic antioxidant properties; POD: peroxidase. Level of significance is ^*^ *P* < 0.05.

To better understand the influence of the tested polysaccharide elicitors on plant defense and secondary metabolite production in shoot cultures, nonenzymatic antioxidant properties (NEAOP) determined by using *β*-carotene-linoleic acid oxidation method as well as enzymatic antioxidant activity represented by POD were examined. NEAOP and POD activity in* H. perforatum *shoot culture after treatment by CHI, PEC, and DEX are shown in Figure 2. An extract that inhibits *β*-carotene bleaching can be described as a free-radical scavenger and a primary antioxidant [67]. In this study, NEAOP was significantly elevated in shoot cultures treated by CHI, PEC, and DEX in the early phase of postelicitation period (day 7), but thereafter (days 14 and 21) NEAOP in treated shoots remained similar to control (Figure 2(a)). A significant negative correlation was observed between NEAOP and hypericin content in elicited shoot culture (*r* = −0.535; ^*^ *P* < 0.05). On the other hand, nonsignificant correlation was found between NEAOP and TP or TF contents. Even if antioxidant activities from different plant sources are usually derived from phenolic-type compounds [68, 69], these effects do not always correlate with the presence of specific phenolics, in particular some subclass of flavonoids or naphtodianthrones. With regard to naphtodianthrones, it was shown that hypericin which has six hydroxyl groups did not demonstrate antioxidant activity, probably due to its hydrophobicity [70].

In this study, POD activity was significantly elevated in shoot cultures treated by PEC during the entire period of postelicitation, while CHI-elicited shoots showed increased POD activity at days 14 and 21, compared to control shoots (Figure 2(b)). It is worth noting that application of both elicitors CHI and PEC significantly increased POD activity at day 14 to a higher level (about 2-fold) than the control shoots. In contrast, addition of DEX to the culture medium had no effect on POD activity in elicited shoots. Our results (Table 1) showed a clear correlation between POD activity and production of TF (*r* = 0.648; ^**^ *P* < 0.01) and hypericin (*r* = 0.550; ^*^ *P* < 0.05). Recent studies reported that peroxidases catalyze the oxidation of organic substrates, including phenolics, in the presence of hydrogen peroxide and have been implicated in the processes of plant growth and development, cell wall formation, and defense responses [71, 72]. Plant* in vitro* culture is likely to be indirectly affected by peroxidases, antioxidative enzymes known to be involved in stress responses [73]. For instance, Santarem et al. [42] demonstrated increased production of TP and hypericin level, followed by a notable decrease in POD activity in* H. perforatum* adventitious shoot cultures treated with* A. rhizogenes*. These authors proposed that metabolic changes may represent a defense strategy of* H. perforatum* against the infection by phytobacteria. On the other hand, Savio et al. [74] showed increased POD activity during the proliferation of* H. perforatum* shoots cultivated in semisolid medium resulting in the reduction of TP levels and hypericin accumulation. Accordingly, the variation in the phenolic levels seemed to be related to the POD activity [74]. Taking these heterogeneous results into account, further research is needed to reveal the possible synergistic or antagonistic relations between the individual components of the complex extract and assess their impact on the antioxidant responses of* H. perforatum *shoot cultures.

## 4. Conclusions

In conclusion, the present work demonstrates that polysaccharides such as chitin, pectin, and dextran differently affected phenylpropanoid and naphtodianthrone production in* H. perforatum* shoot cultures. Polysaccharide elicitors tested here inhibited flavonoid production due to suppressed CHFI activity, while induction of PAL activity probably triggers an alternative naphtodianthrone pathway. Elicited shoots synthesized and stored significant quantities of hypericin and pseudohypericin indicating that naphtodianthrone biosynthesis is prioritized over phenylpropanoids. The results for antioxidant activity of* H. perforatum* shoots indicated that the lower response of nonenzymatic antioxidant properties to polysaccharide treatment in early phase of postelicitation may be compensated by the increased peroxidase activity during the long-term cultivation. The specific and diverse effects of polysaccharide elicitors, as observed in this study, indicated a modification of the accumulation of secondary metabolites with antioxidant properties. The investigation of interrelationship between phenylpropanoid and naphtodianthrone productions with antioxidant activity will be a promising field to understand and elucidate possible mechanisms for utilization of* H. perforatum* as sources of bioactive compounds in food and pharmaceutical industry.

## Figures and Tables

**Table 1:** Correlation analysis between secondary metabolite productions and antioxidant activities in *Hypericum perforatum* shoot cultures.

*r*	TP	TF	PAL	CHFI	HYP	PHYP	NEAOP
TF	0.571^*^						
PAL	NS	NS					
CHFI	NS	NS	NS				
HYP	NS	NS	0.547^*^	NS			
PHYP	NS	NS	0.563^*^	NS	0.854^***^		
NEAOP	NS	NS	NS	NS	−0.535^*^	NS	
POD	NS	0.648^**^	NS	−0.783^***^	0.550^*^	NS	NS

*r*: Pearson's coefficient; TP: total phenolics; TF: total flavonoids; PAL: phenylalanine ammonia lyase; CHFI: chalcone flavanone isomerase; HYP: hypericin; PHYP: pseudohypericin; NEAOP: nonenzymatic antioxidant property; POD: peroxidase. Levels of significance are ^*^ *P* < 0.05, ^**^ *P* < 0.01, and ^***^ *P* < 0.001. NS indicates nonsignificant values.

## Abbreviations

CE: Catechin equivalents
CHFI: Chalcone-flavanone isomerase
CHI: Chitin
DEX: Dextran
DM: Dry mass
GAE: Gallic acid equivalents
NEAOP: Nonenzymatic antioxidant properties
PAL: Phenylalanine ammonia lyase
PEC: Pectin
POD: Peroxidase
TF: Total flavonoids
TP: Total phenolics.

## References

[1] Nahrstedt A., Butterweck V. (2010). Lessons learned from herbal medicinal products: the example of St. John's wort. *Journal of Natural Products*.

[2] Süntar I. P., Akkol E. K., Yilmazer D., Baykal T., Kirmizibekmez H., Alper M., Yeşilada E. (2010). Investigations on the *in vivo* wound healing potential of *Hypericum perforatum* L. *Journal of Ethnopharmacology*.

[3] Sosa S., Pace R., Bornancin A., Morazzoni P., Riva A., Tubaro A., Della Loggia R. (2007). Topical anti-inflammatory activity of extracts and compounds from *Hypericum perforatum* L.. *Journal of Pharmacy and Pharmacology*.

[4] Quiney C., Billard C., Faussat A.-M., Salanoubat C., Kolb J.-P. (2007). Hyperforin inhibits P-gp and BCRP activities in chronic lymphocytic leukaemia cells and myeloid cells. *Leukemia and Lymphoma*.

[5] Jacobson J. M., Feinman L., Liebes L., Ostrow N., Koslowski V., Tobia A., Cabana B. E., Lee D.-H., Spritzler J., Prince A. M. (2001). Pharmacokinetics, safety, and antiviral effects of hypericin, a derivative of St. John's wort plant, in patients with chronic hepatitis C virus infection. *Antimicrobial Agents and Chemotherapy*.

[6] Saddiqe Z., Naeem I., Maimoona A. (2010). A review of the antibacterial activity of *Hypericum perforatum* L.. *Journal of Ethnopharmacology*.

[7] Silva B. A., Malva J. O., Dias A. C. P. (2008). St. John's wort (*Hypericum perforatum*) extracts and isolated phenolic compounds are effective antioxidants in several *in vitro* models of oxidative stress. *Food Chemistry*.

[8] Schempp C. M., Simon-Haarhaus B., Simon J. C. (2002). Phototoxic and apoptosis-inducing capacity of pseudohypericin. *Planta Medica*.

[9] Medina M. A., Martínez-Poveda B., Amores-Sánchez M. I., Quesada A. R. (2006). Hyperforin: more than an antidepressant bioactive compound?. *Life Sciences*.

[10] Sarris J., Fava M., Schweitzer I., Mischoulon D. (2012). St. John's wort (*Hypericum perforatum*) versus sertraline and placebo in major depressive disorder: continuation data from a 26-week RCT. *Pharmacopsychiatry*.

[11] Barnes J., Anderson L. A., Phillipson J. D. (2001). St. John's wort (*Hypericum perforatum* L.): a review of its chemistry, pharmacology and clinical properties. *Journal of Pharmacy and Pharmacology*.

[12] Büter B., Orlacchio C., Soldati A., Berger K. (1998). Significance of genetic and environmental aspects in the field cultivation of *Hypericum perforatum*. *Planta Medica*.

[13] Kirakosyan A., Sirvent T. M., Gibson D. M., Kaufman P. B. (2004). The production of hypericins and hyperforin by *in vitro* cultures of St. John's wort (*Hypericum perforatum*). *Biotechnology and Applied Biochemistry*.

[14] Zobayed S., Saxena P. K. (2004). Production of St. John's wort plants under controlled environment for maximizing biomass and secondary metabolites. *In Vitro Cellular & Developmental Biology-Plant*.

[15] Kopleman S. H., Augsburger L. L., NguyenPho A., Zito W. S., Muller F. X. (2001). Selected physical and chemical properties of commercial *Hypericum perforatum* extracts relevant for formulated product quality and performance. *AAPS PharmSci*.

[16] Murch S. J., Saxena P. K. (2006). St. John's wort (*Hypericum perforatum* L.): challenges and strategies for production of chemically consistent plants. *Canadian Journal of Plant Science*.

[17] Murthy H. N., Lee E.-J., Paek K.-Y. (2014). Production of secondary metabolites from cell and organ cultures: strategies and approaches for biomass improvement and metabolite accumulation. *Plant Cell, Tissue and Organ Culture*.

[18] Ramachandra Rao S., Ravishankar G. A. (2002). Plant cell cultures: chemical factories of secondary metabolites. *Biotechnology Advances*.

[19] Zhao J., Davis L. C., Verpoorte R. (2005). Elicitor signal transduction leading to production of plant secondary metabolites. *Biotechnology Advances*.

[20] Dörnenburg H. (2004). Evaluation of immobilisation effects on metabolic activities and productivity in plant cell processes. *Process Biochemistry*.

[21] Namdeo A. G. (2007). Plant cell elicitation for production of secondary metabolites: a review. *Pharmacognosy Reviews*.

[22] Weathers P. J., Towler M. J., Xu J. (2010). Bench to batch: advances in plant cell culture for producing useful products. *Applied Microbiology and Biotechnology*.

[23] Komaraiah P., Reddy G. V., Reddy P. S., Raghavendra A. S., Ramakrishna S. V., Reddanna P. (2003). Enhanced production of antimicrobial sesquiterpenes and lipoxygenase metabolites in elicitor-treated hairy root cultures of *Solanum tuberosum*. *Biotechnology Letters*.

[24] Zhao J.-L., Zhou L.-G., Wu J.-Y. (2010). Effects of biotic and abiotic elicitors on cell growth and tanshinone accumulation in *Salvia miltiorrhiza* cell cultures. *Applied Microbiology and Biotechnology*.

[25] Cai Z., Kastell A., Mewis I., Knorr D., Smetanska I. (2012). Polysaccharide elicitors enhance anthocyanin and phenolic acid accumulation in cell suspension cultures of *Vitis vinifera*. *Plant Cell, Tissue and Organ Culture*.

[26] Lim F. L., Yam M. F., Asmawi M. Z., Chan L.-K. (2013). Elicitation of *Orthosiphon stamineus* cell suspension culture for enhancement of phenolic compounds biosynthesis and antioxidant activity. *Industrial Crops and Products*.

[27] Kirakosyan A., Hayashi H., Inoue K., Charchoglyan A., Vardapetyan H. (2000). Stimulation of the production of hypericins by mannan in *Hypericum perforatum* shoot cultures. *Phytochemistry*.

[28] Sirvent T., Gibson D. (2002). Induction of hypericins and hyperforin in *Hypericum perforatum* L. in response to biotic and chemical elicitors. *Physiological and Molecular Plant Pathology*.

[29] Walker T. S., Pal Bais H., Vivanco J. M. (2002). Jasmonic acid-induced hypericin production in cell suspension cultures of *Hypericum perforatum* L. (St. John's wort). *Phytochemistry*.

[30] Gadzovska S., Maury S., Ounnar S., Righezza M., Kascakova S., Refregiers M., Spasenoski M., Joseph C., Hagège D. (2005). Identification and quantification of hypericin and pseudohypericin in different *Hypericum perforatum* L. *in vitro* cultures. *Plant Physiology and Biochemistry*.

[31] Vardapetyan H. R., Oganesyan A. A., Kabasakalyan E. E., Tiratsuyan S. G. (2006). The influence of some elicitors on growth and morphogenesis of *Hypericum perforatum* L. callus cultures. *Russian Journal of Developmental Biology*.

[32] Gadzovska S., Maury S., Delaunay A., Spasenoski M., Joseph C., Hagège D. (2007). Jasmonic acid elicitation of *Hypericum perforatum* L. cell suspensions and effects on the production of phenylpropanoids and naphtodianthrones. *Plant Cell, Tissue and Organ Culture*.

[33] Gadzovska-Simic S., Tusevski O., Antevski S., Atanasova-Pancevska N., Petreska J., Stefova M., Kungulovski D., Spasenoski M. (2012). Secondary metabolite production in *Hypericum perforatum* L. cell suspensions upon elicitation with fungal mycelia from *Aspergillus flavus*. *Archives of Biological Sciences*.

[34] Gadzovska S., Maury S., Delaunay A., Spasenoski M., Hagège D., Courtois D., Joseph C. (2013). The influence of salicylic acid elicitation of shoots, callus, and cell suspension cultures on production of naphtodianthrones and phenylpropanoids in *Hypericum perforatum* L.. *Plant Cell, Tissue and Organ Culture*.

[35] Murashige T., Skoog F. (1962). A revised medium for rapid growth and bioassays with tobacco tissue cultures. *Physiologia Plantarum*.

[36] Gamborg O. L., Miller R. A., Ojima K. (1968). Nutrient requirements of suspension cultures of soybean root cells. *Experimental Cell Research*.

[37] Komaraiah P., Naga Amrutha R., Kavi Kishor P. B., Ramakrishna S. V. (2002). Elicitor enhanced production of plumbagin in suspension cultures of *Plumbago rosea* L.. *Enzyme and Microbial Technology*.

[38] Singleton V. L., Rossi J. A. (1965). Colorimetry of total phenolics with phosphomolybdic-phosphotungstic acid reagents. *The American Journal of Enology and Viticulture*.

[39] Makris D. P., Boskou G., Andrikopoulos N. K. (2007). Polyphenolic content and in vitro antioxidant characteristics of wine industry and other agri-food solid waste extracts. *Journal of Food Composition and Analysis*.

[40] Marron N., Delay D., Petit J.-M., Dreyer E., Kahlem G., Delmotte F. M., Brignolas F. (2002). Physiological traits of two *Populus* x *euramericana* clones, Luisa Avanzo and Dorskamp, during a water stress and re-watering cycle. *Tree Physiology*.

[41] González L. F., Rojas M. C., Perez F. J. (1999). Diferulate and lignin formation is related to biochemical differences of wall-bound peroxidases. *Phytochemistry*.

[42] Santarem E. R., Zamban D. C., Felix L. M., Astarita L. V. (2008). Secondary metabolism of *Hypericum perforatum* induced by *Agrobacterium rhizogenes*. *In Vitro Cellular & Developmental Biology: Animal*.

[43] Cui X.-H., Murthy H. N., Wu C.-H., Paek K.-Y. (2010). Sucrose-induced osmotic stress affects biomass, metabolite, and antioxidant levels in root suspension cultures of *Hypericum perforatum* L.. *Plant Cell, Tissue and Organ Culture*.

[44] Dixon R. A., Achnine L., Kota P., Liu C.-J., Reddy M. S. S., Wang L. (2002). The phenylpropanoid pathway and plant defence—a genomics perspective. *Molecular Plant Pathology*.

[45] Tusevski O., Petreska Stanoeva J., Stefova M., Gadzovska Simic S. (2014). *Agrobacterium* enhances xanthone production in *Hypericum perforatum* cell suspensions. *Plant Growth Regulation*.

[46] Bhuiyan M. N. H., Adachi T. (2003). Stimulation of betacyanin synthesis through exogenous methyl jasmonate and other elicitors in suspension-cultured cells of *Portulaca*. *Journal of Plant Physiology*.

[47] Fang Y., Smith M. A. L., Pépin M.-F. (1999). Effects of exogenous methyl jasmonate in elicited anthocyanin-producing cell cultures of ohelo (*Vaccinium pahalae*). *In Vitro Cellular & Developmental Biology:P Plant*.

[48] Conceição L. F. R., Ferreres F., Tavares R. M., Dias A. C. P. (2006). Induction of phenolic compounds in *Hypericum perforatum* L. cells by *Colletotrichum gloeosporioides* elicitation. *Phytochemistry*.

[49] Kartnig T., Göbel I., Heydel B. (1996). Production of hypericin, pseudohypericin and flavonoids in cell cultures of various *Hypericum* species and their chemotypes. *Planta Medica*.

[50] Pasqua G., Avato P., Monacelli B., Santamaria A. R., Argentieri M. P. (2003). Metabolites in cell suspension cultures, calli, and in vitro regenerated organs of *Hypericum perforatum* cv. Topas. *Plant Science*.

[51] Tusevski O., Petreska Stanoeva J., Stefova M., Pavokovic D., Gadzovska S. (2014). Identification and quantification of phenolic compounds in *Hypericum perforatum* L. transgenic shoots. *Acta Physiologiae Plantarum*.

[52] Liu X.-N., Zhang X.-Q., Zhang S.-X., Sun J.-S. (2007). Regulation of metabolite production by precursors and elicitors in liquid cultures of *Hypericum perforatum*. *Plant Cell, Tissue and Organ Culture*.

[53] Karppinen K., Hohtola A. (2008). Molecular cloning and tissue-specific expression of two cDNAs encoding polyketide synthases from *Hypericum perforatum*. *Journal of Plant Physiology*.

[54] Falk H. (1999). From the photosensitizer hypericin to the photoreceptor stentorin—the chemistry of phenantroperylene quinones. *Angewandte Chemie*.

[55] Bais H. P., Vepachedu R., Lawrence C. B., Stermitz F. R., Vivanco J. M. (2003). Molecular and biochemical characterization of an enzyme responsible for the formation of hypericin in St. John's wort (*Hypericum perforatum* L.). *Journal of Biological Chemistry*.

[56] Košuth J., Katkovčinová Z., Olexová P., Čellárová E. (2007). Expression of the hyp-1 gene in early stages of development of *Hypericum perforatum* L. *Plant Cell Reports*.

[57] Karppinen K. (2010). Biosynthesis of hypericins and hyperforins in *Hypericum perforatum* L. (St. John’s wort)—precursors and genes involved. *Acta Universitatis Ouluensis A: Scientiae Rerum Naturalium*.

[58] Michalska K., Fernandes H., Sikorski M., Jaskolski M. (2010). Crystal structure of Hyp-1, a St. John's wort protein implicated in the biosynthesis of hypericin. *Journal of Structural Biology*.

[59] Xu M.-J., Dong J.-F., Zhang G. (2005). Enhancement of hypericin production and cell growth of *Hypericum perforatum* L. suspension cultures by nitric oxide. *Chinese journal of biotechnology*.

[60] Klejdus B., Kováčik J., Babula P. (2013). PAL inhibitor evokes different responses in two *Hypericum species*. *Plant Physiology and Biochemistry*.

[61] Han Y.-S., van der Heijden R., Verpoorte R. (2001). Biosynthesis of anthraquinones in cell cultures of the Rubiaceae. *Plant Cell, Tissue and Organ Culture*.

[62] Pillai P. P., Nair A. R. (2014). Hypericin biosynthesis in *Hypericum hookerianum* Wight and Arn: investigation on biochemical pathways using metabolite inhibitors and suppression subtractive hybridization. *Comptes Rendus Biologies*.

[63] Dixon R. A., Paiva N. L. (1995). Stress-induced phenylpropanoid metabolism. *Plant Cell*.

[64] Ververidis F., Trantas E., Douglas C., Vollmer G., Kretzschmar G., Panopoulos N. (2007). Biotechnology of flavonoids and other phenylpropanoid-derived natural products. Part I. Chemical diversity, impacts on plant biology and human health. *Biotechnology Journal*.

[65] Hillwig M. L., Hammer K. D. P., Birt D. F., Wurtele E. S. (2008). Characterizing the metabolic fingerprint and anti-inflammatory activity of *Hypericum gentianoides*. *Journal of Agricultural and Food Chemistry*.

[66] Kusari S., Zühlke S., Borsch T., Spiteller M. (2009). Positive correlations between hypericin and putative precursors detected in the quantitative secondary metabolite spectrum of *Hypericum*. *Phytochemistry*.

[67] Liyana-Pathirana C. M., Shahidi F. (2006). Antioxidant properties of commercial soft and hard winter wheats (*Triticum aestivum* L.) and their milling fractions. *Journal of the Science of Food and Agriculture*.

[68] Cai Y., Luo Q., Sun M., Corke H. (2004). Antioxidant activity and phenolic compounds of 112 traditional Chinese medicinal plants associated with anticancer. *Life Sciences*.

[69] Bozin B., Mimica-Dukic N., Samojlik I., Goran A., Igic R. (2008). Phenolics as antioxidants in garlic (*Allium sativum* L., Alliaceae). *Food Chemistry*.

[70] Conforti F., Statti G. A., Tundis R., Menichini F., Houghton P. (2002). Antioxidant activity of methanolic extract of *Hypericum triquetrifolium* Turra aerial part. *Fitoterapia*.

[71] Hatzilazarou S. P., Syros T. D., Yupsanis T. A., Bosabalidis A. M., Economou A. S. (2006). Peroxidases, lignin and anatomy during in vitro and ex vitro rooting of gardenia (*Gardenia jasminoides* Ellis) microshoots. *Journal of Plant Physiology*.

[72] Abbasi B. H., Khan M., Guo B., Bokhari S. A., Khan M. A. (2011). Efficient regeneration and antioxidative enzyme activities in *Brassica rapa* var. *turnip*. *Plant Cell, Tissue and Organ Culture*.

[73] Kormutak A., Vookova B. (2001). Peroxidase activity in non-embryogenic and embryogenic calli and in developing somatic embryos of white fir (*Abies concolor* Gord. et Glend). *Plant Biosystems*.

[74] Savio L. E. B., Astarita L. V., Santarém E. R. (2012). Secondary metabolism in micropropagated *Hypericum perforatum* L. grown in non-aerated liquid medium. *Plant Cell, Tissue and Organ Culture*.

